# Precision Oncology: Cancer Specialists’ Perspectives on Molecular Profiling, Targeted Therapy, and Barriers to Access

**DOI:** 10.14740/jocmr6739

**Published:** 2026-10-23

**Authors:** Pushpita Roy, Dipaloke Banik, Syed Md. Asadul Hoque, Mahfuz Hasan Khan

**Affiliations:** a College of Medicine and Veterinary Medicine, Deanery of Clinical Sciences, Edinburgh Medical School, The University of Edinburgh, Edinburgh, UK; b School of Public Health, SUNY Downstate Health Sciences University, Brooklyn, NY, USA; c Department of Radiotherapy, Dhaka Medical College and Hospital, Dhaka, Bangladesh; d Department of Chemotherapy, Labaid Cancer Hospital and Super Specialty Center, Dhaka, Bangladesh

**Keywords:** Precision oncology, Molecular profiling, Targeted therapy, Barriers, Bangladesh, Oncologists, Cancer specialists, Clinical practice

## Abstract

**Background:**

Precision oncology has transformed cancer care through molecular profiling and biomarker-guided therapies. However, access remains uneven in resource-constrained settings. In Bangladesh, quantitative evidence characterizing barriers to molecular profiling and targeted therapy remains limited, particularly regarding barriers beyond financial constraints. This study aims to describe oncologists’ perspective regarding current molecular profiling practices and receipt of targeted therapy among eligible patients, and to identify major financial and non-financial barriers and potential strategies for improving access to precision oncology as perceived by the physicians.

**Methods:**

A nationwide cross-sectional survey was conducted from June to July 2026 among cancer specialists actively involved in cancer care in Bangladesh. A structured questionnaire assessed molecular profiling and targeted therapy practices, perceived barriers, and potential strategies for improving access. Data from 52 respondents were analyzed descriptively using frequencies and percentages.

**Results:**

Molecular profiling was recommended often or always by 27 (51.9%) respondents. estrogen receptor/progesterone receptor/human epidermal growth factor receptor 2 (ER/PR/HER2) (90.4%), microsatellite instability/mismatch repair (MSI/MMR) (82.7%), and epidermal growth factor receptor (EGFR) (80.8%) were the most requested molecular tests. However, 45 (86.5%) respondents reported that no more than 25% of eligible patients ultimately received targeted therapy. High cost of targeted therapy was rated as a major or very major barrier by 46 (88.5%) respondents, followed by high cost of molecular testing (45, 86.5%). Among non-financial barriers, limited testing facilities (29, 55.8%), limited patient understanding or acceptance of molecular testing (28, 53.8%), and long turnaround time for molecular testing results (25, 48.1%) were most frequently rated as major or very major barriers. Most respondents (42, 80.8%) agreed or strongly agreed that targeted therapy should become standard practice in Bangladesh where indicated. Reduced testing costs (43, 82.7%) and government-subsidized insurance coverage (33, 63.5%) were the most frequently selected strategies for improving access.

**Conclusion:**

Cancer specialists in Bangladesh reported substantial use of molecular profiling but limited receipt of targeted therapy among eligible patients. Although financial constraints emerged as the most prominent barriers, important non-financial challenges related to testing infrastructure, patient understanding and acceptance, and turnaround time were also identified. In the context of limited existing quantitative evidence from Bangladesh, these findings provide initial insight into the relative prominence of financial and non-financial barriers and may inform strategies to improve access to precision oncology.

## Introduction

Globally, cancer is the second leading cause of death, with low- and middle-income countries contributing substantially to cancer-related mortality [1, 2]. Between 2018 and 2022, cancer incidence in Bangladesh increased by 11%, while cancer-related mortality rose by 8% [3], highlighting the growing burden of cancer as a major non-communicable disease despite substantial global advances in molecular oncology, cancer genomics, and precision oncology.

Precision oncology uses molecular tumor characterization to guide personalized treatment, with the principle of delivering the right treatment to the right patient at the right dose and time [4]. In this study, molecular profiling refers to testing tumor tissue, blood, or other biological samples for molecular characteristics, including genes, proteins, and other biomarkers, that may inform cancer diagnosis, prognosis, or treatment selection [5, 6]. Targeted therapy refers to anticancer treatment directed against specific molecular targets or pathways involved in cancer growth and survival [7, 8].

Advances in genomics, omics technologies, and molecular biology have increasingly enabled such biomarker-guided approaches to cancer care. However, their implementation remains limited in developing countries like Bangladesh. Beyond economic constraints, barriers may include inadequate diagnostic infrastructure, health-system and policy limitations, provider-related challenges, and patient-related factors.

Previous studies have explored barriers and broader challenges affecting cancer care in Bangladesh [9–12]. A regional study across SAARC countries identified inadequate healthcare infrastructure, limited financial resources, cultural barriers, and poor awareness of cancer prevention and treatment as major challenges to cancer care in those countries [13]. More recently, gaps in the cancer care system in Bangladesh have been highlighted alongside proposed strategies such as a national cancer registry and integration of artificial intelligence [3]. However, despite this growing evidence on broader cancer-care challenges, barriers specifically affecting the implementation of precision oncology in Bangladesh remain largely unexplored. To our knowledge, no previous Bangladeshi study has quantitatively evaluated the barriers to incorporating molecular profiling and biomarker-guided targeted therapies into routine cancer care perceived by the oncologists.

The present survey therefore aimed to identify the perceived barriers to molecular profiling and targeted therapy among cancer specialists in Bangladesh and to determine the interventions they consider most important for improving access to precision oncology. These findings may help inform future healthcare planning, resource prioritization, and policy development for implementation of precision oncology in the management of cancer patients in Bangladesh.

## Materials and Methods

### Study design

This was a cross-sectional survey of cancer specialists in Bangladesh conducted from June through July 2026 to assess perceived barriers to molecular profiling and targeted therapy. The STROBE reporting guideline for cross-sectional studies was followed [14].

### Ethical considerations

The study involved no direct interaction or intervention with human participants and did not involve experimentation on humans, animals, or any other living species. Participation in the questionnaire-based survey was entirely voluntary, and participants were free to decline or discontinue participation at any time. No patient-level information or biological samples were collected, and no intervention was performed as part of the study. Participant responses were collected anonymously and used solely for research purposes, with confidentiality maintained throughout the study.

### IRB approval

Ethical review was sought to ensure appropriate research oversight, and approval was obtained from the Institutional Review Board (IRB) of Labaid Cancer Hospital and Super Specialty Centre, Dhaka, Bangladesh, prior to the commencement of the study and data collection.

### Questionnaire development

A structured questionnaire was developed to assess cancer specialists’ perceptions regarding barriers to molecular profiling and targeted therapy in Bangladesh. The survey instrument consisted of 19 items grouped into four domains: (1) respondent demographics and practice characteristics, including specialty, years of practice, primary role, and healthcare setting; (2) current utilization of molecular profiling, including frequency of test recommendation, commonly requested molecular assays, and utilization of targeted therapies; (3) perceived barriers to molecular testing and targeted therapy; and (4) potential interventions to improve access to precision oncology services (Table 1).

**Table 1:** Survey Questionnaire and Response Options

Domain	Survey question/item	Response options
Participant characteristics	1. What is your primary specialty?	Clinical Oncology; Radiation Oncology; Surgical Oncology; Medical Oncology; Hematology; Other (Please specify)
	2. Years in independent clinical practice	< 5 years; 5–10 years; 11–20 years; > 20 years
	3. Identify your primary role	Clinician; Educator; Researcher
	4. Which of the following is your current primary practice setting?	Government Hospital; University Hospital; Private Hospital; Autonomous nonprofit Institution/Hospital
	5. Which level of healthcare facility best describes your current primary practice setting?	Primary care hospital/center; Secondary-level hospital; Tertiary referral hospital; Specialized cancer center; Multiple practice settings; Other (please specify)
	6. Which of the following is the approximate number of cancer patients managed by you per month?	< 50; 50–100; 100–200; > 200
Molecular profiling and targeted therapy practices	7. How often do you recommend molecular profiling (genomic/biomarker testing) for eligible cancer patients?	Never; Rarely (less than 25% of cases); Sometimes (25–50% of cases); Often (more than 50% of cases); Always
	8. Which molecular tests do you commonly request? (Select all that apply)	ER, PR, HER2; EGFR; ALK; ROS1; KRAS/BRAF; MSI/MMR; PD-L1; NGS Panel; BRCA1/BRCA2; Other (Please specify)
	9. Approximately what proportion of your patients who are eligible ultimately receive targeted therapy?	< 10%; 10–25%; 26–50%; 51–75%; > 75%
Perceived barriers	10. High cost of molecular testing	1 = Not a barrier to 5 = Very major barrier
	11. High cost of targeted therapy	1 = Not a barrier to 5 = Very major barrier
	12. Limited availability of testing facilities within Bangladesh	1 = Not a barrier to 5 = Very major barrier
	13. Limited availability of targeted therapy/supply issues	1 = Not a barrier to 5 = Very major barrier
	14. Long turnaround time for molecular testing results	1 = Not a barrier to 5 = Very major barrier
	15. Limited physician awareness/training regarding molecular-guided treatment	1 = Not a barrier to 5 = Very major barrier
	16. Limited local clinical guideline	1 = Not a barrier to 5 = Very major barrier
	17. Limited patient understanding or acceptance of molecular testing	1 = Not a barrier to 5 = Very major barrier
Attitudes and potential interventions	18. Targeted therapy should become standard practice in Bangladesh where indicated.	1 = Strongly disagree to 5 = Strongly agree
	19. Which intervention would MOST improve access to molecular profiling and targeted therapy? (Choose two)	Reduced testing cost; Government-subsidized insurance coverage; More local laboratories; Physician education; Faster reporting; Increased drug availability

ALK: anaplastic lymphoma kinase; BRCA1/BRCA2: breast cancer susceptibility gene 1/2; EGFR: epidermal growth factor receptor; ER: estrogen receptor; HER2: human epidermal growth factor receptor 2; KRAS/BRAF: Kirsten rat sarcoma viral oncogene homolog/B-Raf proto-oncogene; MMR: mismatch repair; MSI: microsatellite instability; NGS: next-generation sequencing; PD-L1: programmed death-ligand 1; PR: progesterone receptor; ROS1: ROS proto-oncogene 1.

The questionnaire included a combination of single-response multiple-choice questions, multiple-response questions, and 5-point Likert-scale items. Perceived barriers were assessed using a 5-point Likert scale ranging from “not a barrier” to “very major barrier.” Participants were also asked to indicate their level of agreement with the statement that targeted therapy should become standard practice in Bangladesh where clinically indicated and to identify interventions that could most effectively improve access to molecular profiling and targeted therapies.

For the survey item assessing the proportion of eligible patients who ultimately received targeted therapy, “eligible patients” referred to patients whom the responding cancer specialist considered clinically eligible for targeted therapy in routine practice. As the study did not collect patient-level clinical data, eligibility was based on physicians’ assessment and was not independently verified by the investigators.

For the item assessing potential strategies to improve access, respondents were asked to select two interventions they considered most likely to improve access to molecular profiling and targeted therapy. As multiple responses were permitted, each intervention was summarized independently using frequencies and percentages, and the percentages were not expected to sum to 100%.

The National Survey of Precision Medicine in Cancer Treatment was thoroughly reviewed during questionnaire development to inform the selection and framing of relevant survey items. The questionnaire was developed by the investigators following a review of published literature and previous physician surveys examining the utilization of molecular/genomic testing, barriers to biomarker-guided cancer care, access to matched targeted therapies, and implementation of precision oncology in resource-constrained settings [15–25].

Prior to dissemination, the questionnaire was independently reviewed by three oncology specialists to assess the relevance, clarity, comprehensibility, and appropriateness of the survey items in the context of cancer care in Bangladesh [26]. Feedback from the expert review was incorporated into the final questionnaire to improve the clarity and content relevance of the survey items.

A link to the questionnaire was distributed via professional email networks, institutional contacts, physician email distribution lists, and oncology-focused WhatsApp groups across Bangladesh. Responses were reviewed for completeness before analysis, and only complete questionnaires from eligible participants were included in the final dataset. The electronic survey platform was configured to restrict each participant to a single submission to minimize duplicate responses.

### Sample size and recruitment period

Given the exploratory and descriptive nature of the study and the specialized population of cancer specialists in Bangladesh, a formal hypothesis-based sample size calculation was not performed. Published literature has previously estimated approximately 250 qualified radiation oncologists and 22 qualified medical oncologists in Bangladesh [27]. However, these figures represent reported estimates for specific oncology specialties and may not reflect the complete or current national oncology workforce. The survey remained open from June to July 2026, during which 52 eligible cancer specialists participated. Considering the relatively limited specialist oncology workforce reported in the literature, the inclusion of 52 cancer specialists represents a considerable number of respondents for an exploratory national survey and was considered appropriate for describing current molecular profiling practices, receipt of targeted therapy among eligible patients, perceived barriers, and potential strategies for improving access.

The questionnaire was distributed through established professional networks, including departmental leadership and institutional representatives, to practicing oncologists working in dedicated cancer centers and major tertiary care hospitals throughout Bangladesh. Eligible participants were physicians currently practicing in Bangladesh who were actively involved in the clinical management of patients with cancer and practicing in an oncology-related specialty. Participation was limited to clinicians working in hospitals, cancer centers, or other clinical oncology settings. The target population comprised cancer specialists practicing in Bangladesh who were actively involved in the clinical management of patients with cancer. Because a comprehensive and up-to-date national sampling frame of all eligible cancer specialists was not available to the investigators, participants were recruited through professional networks and institutional contacts using non-probability convenience sampling.

Eligible participants were invited to complete the questionnaire electronically via Google Forms and were encouraged to share the survey with eligible colleagues working in oncology-related specialties across diverse healthcare settings to maximize study reach and representation. A reminder was sent 2 weeks after the initial invitation to maximize the response rate. No financial incentives were provided for participation.

Because the survey was disseminated through professional networks and institutional contacts and the total number of eligible specialists who received or viewed the invitation could not be reliably determined, a conventional response rate could not be calculated. A total of 52 eligible cancer specialists completed the survey and were included in the analysis.

As the study did not collect patient-level clinical data, eligibility and targeted therapy receipt were based on physicians’ assessments and estimates and were not independently verified by the investigators.

Participation was voluntary, and respondents were informed about the study objectives before providing consent to participate. The survey was administered anonymously, and no directly identifying personal information was collected from participants. Survey responses were analyzed and reported at the group level without identifying individual respondents. The electronic survey platform was configured to restrict each participant to a single submission, thereby minimizing the possibility of duplicate responses.

## Results

A total of 52 cancer specialists participated in the survey. Clinical oncology was the predominant specialty, accounting for 36 (69.2%) respondents, followed by radiation oncology and hematology, with five (9.6%) respondents each, medical oncology with four (7.7%), and surgical oncology with two (3.8%). Most participants had fewer than 5 years of independent clinical practice (31, 59.6%), while 15 (28.8%) had 5–10 years, four (7.7%) had 11–20 years, and two (3.8%) had more than 20 years of experience. All participants identified their primary role as clinicians. Government hospitals were the most common primary practice setting (32, 61.5%), followed by private hospitals (14, 26.9%), autonomous nonprofit institutions/hospitals (4, 7.7%), and university hospitals (2, 3.8%). Nearly half of the respondents practiced in specialized cancer centers (24, 46.2%), while 17 (32.7%) practiced in tertiary referral hospitals. Regarding clinical workload, 20 (38.5%) respondents managed fewer than 50 cancer patients per month, 16 (30.8%) managed 50–100, six (11.5%) managed 100–200, and 10 (19.2%) managed more than 200 patients per month (Table 2).

**Table 2:** Demographics and Characteristics of the Study Participants

Characteristic	n (%)
Primary specialty	
Clinical Oncology	36 (69.2)
Hematology	5 (9.6)
Medical Oncology	4 (7.7)
Radiation Oncology	5 (9.6)
Surgical Oncology	2 (3.8)
Years in independent clinical practice	
< 5 years	31 (59.6)
5–10 years	15 (28.8)
11–20 years	4 (7.7)
> 20 years	2 (3.8)
Primary practice setting	
Government hospital	32 (61.5)
Private hospital	14 (26.9)
Autonomous nonprofit institution/hospital	4 (7.7)
University hospital	2 (3.8)
Level of healthcare facility	
Primary care hospital/center	1 (1.9)
Secondary-level hospital	3 (5.8)
Tertiary referral hospital	17 (32.7)
Specialized cancer center	24 (46.2)
Multiple practice settings	6 (11.5)
Other	1 (1.9)
Approximate number of cancer patients managed per month	
< 50	20 (38.5)
50–100	16 (30.8)
100–200	6 (11.5)
> 200	10 (19.2)

Molecular profiling was recommended with varying frequency among respondents. Eighteen (34.6%) reported recommending molecular profiling often (> 50% of eligible cases), while nine (17.3%) reported always recommending it; together, these represented 27 (51.9%) respondents (Table 3). Seventeen (32.7%) recommended profiling sometimes (25–50% of cases), and eight (15.4%) did so rarely (< 25% of cases); none reported never recommending molecular profiling (Table 3). Estrogen receptor/progesterone receptor/human epidermal growth factor receptor 2 (ER/PR/HER2) testing was the most commonly requested molecular test (47, 90.4%), followed by microsatellite instability/mismatch repair (MSI/MMR) (43, 82.7%), epidermal growth factor receptor (EGFR) (42, 80.8%), programmed death-ligand 1 (PD-L1) (33, 63.5%), next-generation sequencing (NGS) panels (28, 53.8%), breast cancer susceptibility gene 1/2 (BRCA1/BRCA2) (26, 50.0%), ROS proto-oncogene 1 (ROS1) (25, 48.1%), anaplastic lymphoma kinase (ALK) (24, 46.2%), and Kirsten rat sarcoma viral oncogene homolog/B-Raf proto-oncogene (KRAS/BRAF) (16, 30.8%); five (9.6%) respondents reported requesting other molecular tests (Fig. 1).

**Table 3:** Survey Summary: Molecular Profiling and Targeted Therapy Practices Among Participating Cancer Specialists (N = 52)

Practice characteristic	n (%)
Frequency of recommending molecular profiling for eligible patients	
Always	9 (17.3)
Often (> 50% of cases)	18 (34.6)
Sometimes (25–50% of cases)	17 (32.7)
Rarely (< 25% of cases)	8 (15.4)
Proportion of eligible patients ultimately receiving targeted therapy	
< 10%	22 (42.3)
10–25%	23 (44.2)
26–50%	6 (11.5)
51–75%	1 (1.9)

**Figure 1:**
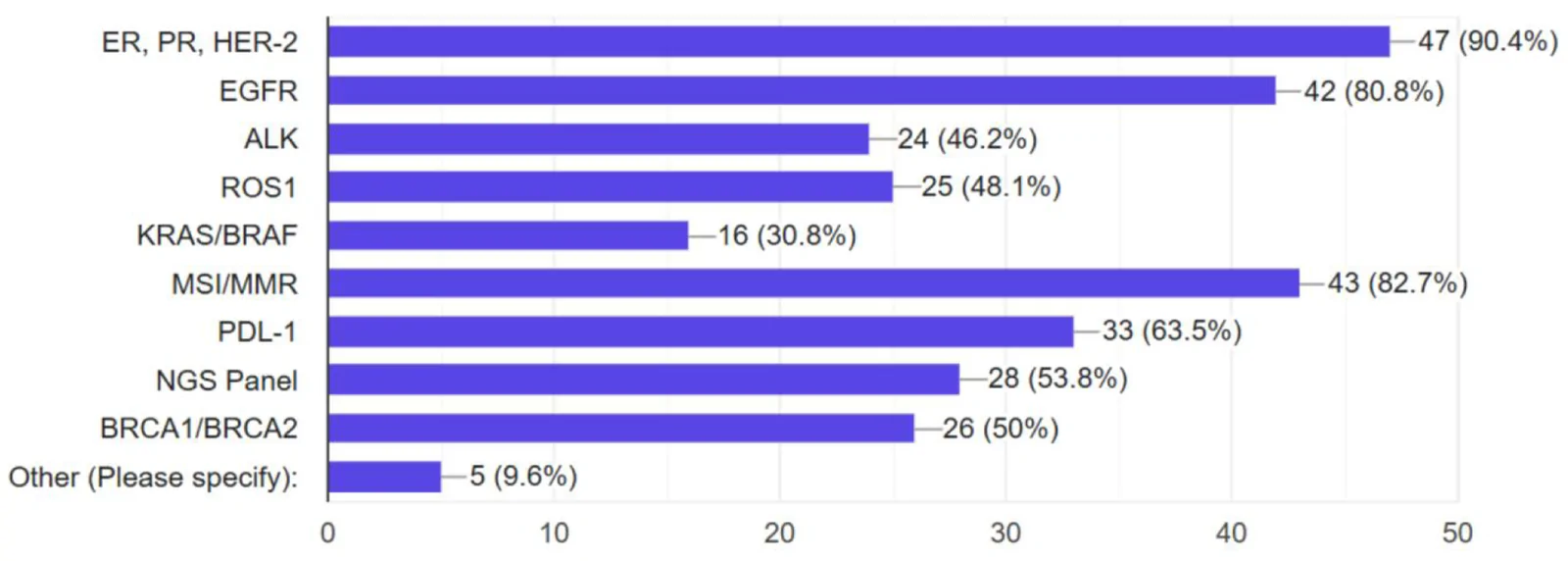
Molecular tests most commonly requested by cancer specialists in Bangladesh. ALK: anaplastic lymphoma kinase; BRCA1/BRCA2: breast cancer susceptibility gene 1/2; EGFR: epidermal growth factor receptor; ER: estrogen receptor; HER-2: human epidermal growth factor receptor 2; KRAS/BRAF: Kirsten rat sarcoma viral oncogene homolog/B-Raf proto-oncogene; MMR: mismatch repair; MSI: microsatellite instability; NGS: next-generation sequencing; PD-L1: programmed death-ligand 1; PR: progesterone receptor; ROS1: ROS proto-oncogene 1.

Despite the reported use of molecular profiling, respondents generally reported low receipt of targeted therapy among eligible patients. Twenty-two (42.3%) estimated that fewer than 10% of their eligible patients ultimately received targeted therapy, while 23 (44.2%) estimated that 10%–25% received it (Table 3). Six (11.5%) reported receipt among 26–50% of eligible patients, and only one (1.9%) reported receipt among 51–75%; no respondent reported that more than 75% of eligible patients received targeted therapy (Table 3). Overall, 45 of 52 respondents (86.5%) reported that no more than 25% of eligible patients ultimately received targeted therapy indicating a substantial clinician-perceived gap between treatment eligibility and targeted therapy receipt (Table 3).

Substantial barriers to molecular profiling and targeted therapy were reported (Table 4). High cost of targeted therapy was the most prominently reported barrier, with 38 (73.1%) respondents rating it as a very major barrier and eight (15.4%) as a major barrier, corresponding to 46 (88.5%) respondents overall. Similarly, the high cost of molecular testing was considered a major or very major barrier by 45 (86.5%) respondents. Both cost-related barriers had a median rating of 5 (interquartile range (IQR), 4–5). Limited availability of testing facilities within Bangladesh was rated as a major or very major barrier by 29 (55.8%), while limited patient understanding or acceptance of molecular testing was reported at these levels by 28 (53.8%). Long turnaround time for molecular testing results was rated as a major or very major barrier by 25 (48.1%) respondents. In comparison, limited local clinical guidelines, limited availability of targeted therapy or supply issues, and limited physician awareness or training regarding molecular-guided treatment were rated as major or very major barriers by 20 (38.5%), 18 (34.6%), and 15 (28.8%) respondents, respectively.

**Table 4:** Perceived Barriers to Molecular Profiling and Targeted Therapy Among Cancer Specialists in Bangladesh (N = 52)

Perceived barrier	Median (IQR)	Rating 4–5, n (%)
High cost of molecular testing	5 (4–5)	45 (86.5)
High cost of targeted therapy	5 (4–5)	46 (88.5)
Limited availability of testing facilities within Bangladesh	4 (3–4)	29 (55.8)
Limited availability of targeted therapy/supply issues	3 (2–4)	18 (34.6)
Long turnaround time for molecular testing results	3 (2–4)	25 (48.1)
Limited physician awareness/training regarding molecular-guided treatment	3 (2–4)	15 (28.8)
Limited local clinical guidelines	3 (2–4)	20 (38.5)
Limited patient understanding or acceptance of molecular testing	4 (3–4)	28 (53.8)

Barrier items were rated on a 5-point scale. Data are presented as median (IQR) and n (%) of respondents assigning a rating of 4 or 5. Higher ratings indicate a greater perceived barrier.

Most respondents expressed support for incorporating targeted therapy into routine cancer care clinically indicated. Thirty-six (69.2%) strongly agreed and six (11.5%) agreed that targeted therapy should become standard practice in Bangladesh where indicated, corresponding to 42 (80.8%) respondents overall. Four (7.7%) were neutral, while one (1.9%) disagreed and five (9.6%) strongly disagreed (Fig. 2).

**Figure 2:**
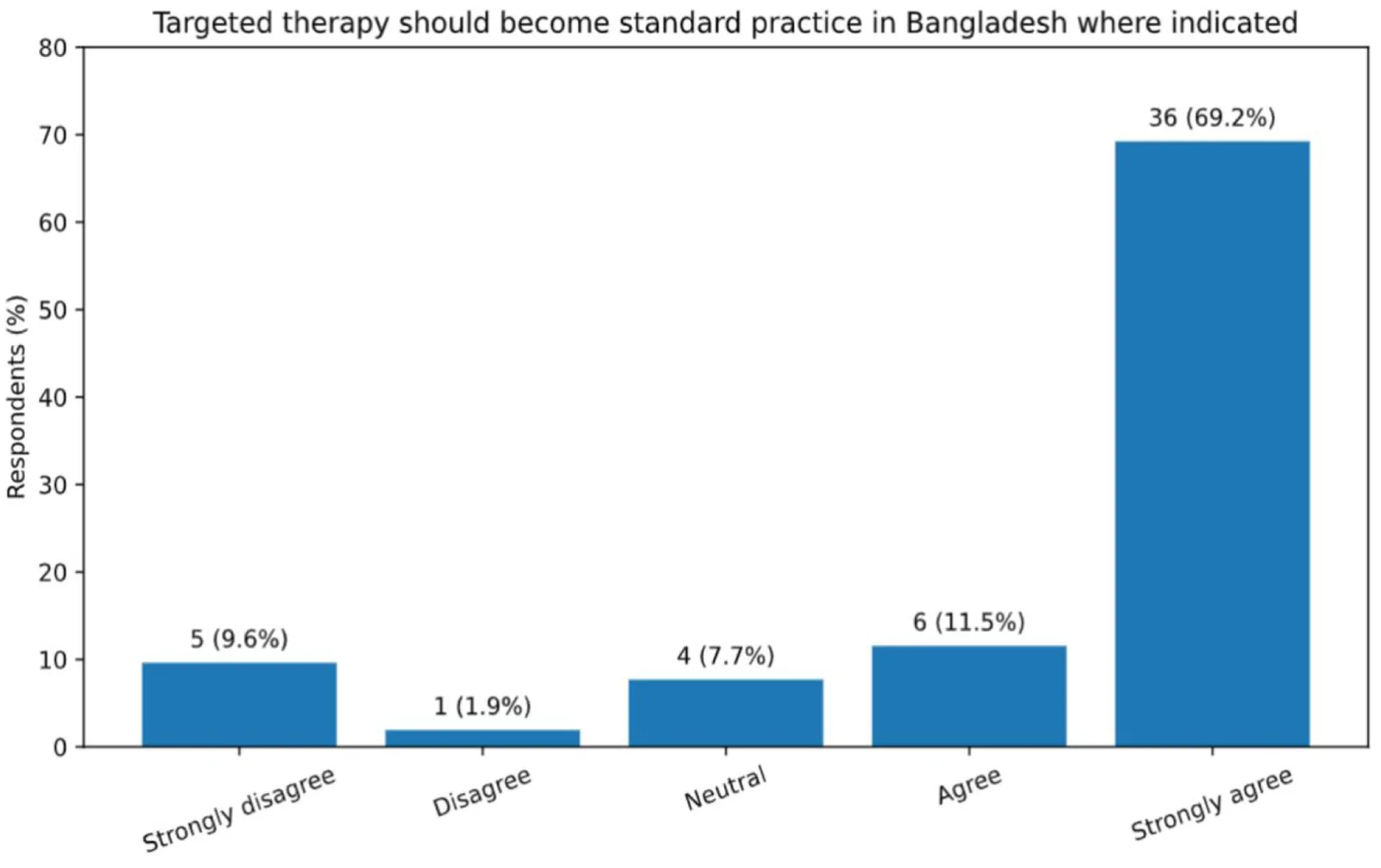
Cancer specialists’ agreement that targeted therapy should become standard practice in Bangladesh where clinically indicated.

When asked to select two interventions that would most improve access to molecular profiling and targeted therapy, reduced testing cost was the most frequently selected measure, reported by 43 (82.7%) respondents, followed by government-subsidized insurance coverage (33, 63.5%). More local laboratories were selected by 15 (28.8%), increased drug availability by 13 (25.0%), and physician education and faster reporting by five (9.6%) respondents each (Fig. 3). These findings indicate that respondents most frequently identified financial interventions, particularly reducing testing costs and expanding insurance support, as measures for improving access to precision oncology services in Bangladesh.

**Figure 3:**
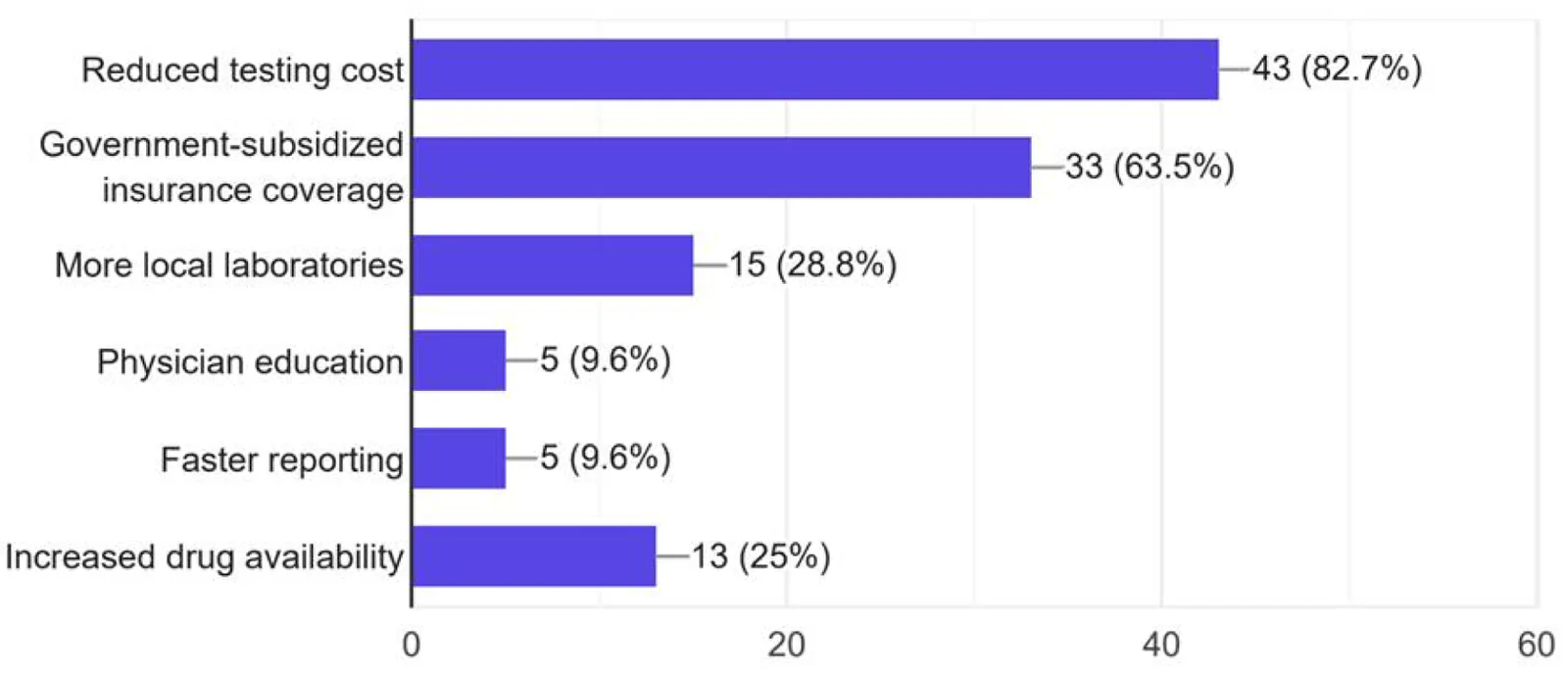
Interventions considered most likely to improve access to molecular profiling and targeted therapy in Bangladesh.

## Discussion

This study provides descriptive insight into the current implementation of molecular profiling and targeted therapy in Bangladesh and, importantly, into barriers that extend beyond financial constraints. More than half of the surveyed cancer specialists reported recommending molecular profiling often or always, and several established biomarkers were commonly incorporated into clinical practice. Nevertheless, 86.5% of respondents estimated that no more than one-quarter of eligible patients ultimately received targeted therapy. This contrast indicates an important gap between the incorporation of molecular diagnostics into clinical practice and the subsequent receipt of molecularly guided treatment. Rather than reflecting a single obstacle, the descriptive pattern suggests that this gap exists within a broader landscape of financial, infrastructural, treatment-access, and patient-level challenges.

The pattern of molecular testing observed in this study indicates that molecular profiling has already become part of oncology practice among the surveyed specialists, although its use varies across biomarkers. ER/PR/HER2, MSI/MMR, and EGFR were the most requested tests, whereas NGS panels and several other molecular assays were reported less frequently. In a multinational survey, 90% of physicians reported using biomarkers, while cost, local availability, and turnaround time were among the major impediments to testing [28]. Greater availability and lower testing costs were among the interventions most frequently identified as likely to increase use [28]. Similarly, an international survey of ASCO members demonstrated considerable geographic variation in genomic testing, with testing costs and access to clinical trials emerging as barriers across regions [29]. These observations are also consistent with more recent literature emphasizing that disparities in precision-oncology diagnostics arise not only from affordability but also from unequal access to genomic technologies and the ability to translate genomic information into appropriate therapies [30]. Thus, the availability of molecular testing alone should not be considered equivalent to effective access to precision oncology.

One of the most notable findings was the low reported receipt of targeted therapy among eligible patients. Although 51.9% of respondents recommended molecular profiling often or always, 42.3% estimated that fewer than 10% of eligible patients ultimately received targeted therapy and another 44.2% estimated receipt among only 10–25%. These descriptive findings do not establish a relationship between testing frequency and treatment receipt, but they demonstrate that relatively frequent molecular profiling can coexist with limited access to molecularly matched treatment. The finding that 86.5% of respondents estimated targeted therapy receipt among no more than one-quarter of their eligible patients suggests a substantial clinician-perceived gap between treatment eligibility and treatment receipt. Importantly, this finding represents physicians’ estimates of their clinical practice rather than patient-level measurement of targeted therapy utilization and should therefore be interpreted as a perceived practice-level access gap. Even in substantially better-resourced settings, oncologists have reported that patients with actionable molecular alterations remain unable to access the corresponding approved targeted therapy [31]. Recent real-world evidence reinforces this distinction: in a study of 653 patients undergoing comprehensive genomic profiling, the overall availability of genome-matched therapy was only 9.5%, and multiple barriers to accessing matched therapy were documented [32]. Similarly, a recent meta-analysis of real-world comprehensive genomic profiling studies found that although actionable alterations were frequently identified, only a minority of patients with actionable findings ultimately received matched targeted therapy [33]. These observations emphasize that the clinical benefit of molecular profiling depends on the ability to translate an actionable result into an accessible treatment.

Financial constraints were the most prominently reported barriers in the present study. High cost of targeted therapy was rated as a major or very major barrier by 88.5% of respondents, while 86.5% reported the same for high cost of molecular testing. A recent hospital-based study estimated an average annual out-of-pocket cancer cost of approximately US$6,504 per patient—more than twice the average annual household income in that study and reported distress financing among approximately 90% of affected households [34]. At the regional level, cancer care across South Asian Association for Regional Cooperation countries has similarly been described as constrained by economic limitations and inadequate infrastructure, with Bangladesh particularly dependent on out-of-pocket expenditure [13]. Against this background, the additional costs associated with molecular diagnostics and targeted agents may create another substantial layer of financial difficulty for patients who are otherwise clinically eligible for precision therapies.

However, an important objective of the present study was to explore major barriers beyond financial constraints, and the descriptive findings demonstrate that affordability does not represent the entire challenge. Limited availability of testing facilities within Bangladesh and limited patient understanding or acceptance of molecular testing were each frequently rated as major or very major barriers, while long turnaround time for molecular testing results was also prominent. These perceptions are consistent with recent assessments of cancer care in Bangladesh describing inadequate cancer-specific molecular genetics infrastructure and the need for some specimens to be sent outside the country for testing [3]. Similar challenges have been described elsewhere in South Asia. A recent survey from Pakistan identified intersecting system-, clinician-, and patient-level barriers to oncology genetic testing, including cost, workflow delays, fragmented processes, limitations in genetics training, and patient misconceptions [35]. More recent literature similarly recognizes that cost is only one dimension of unequal access to precision oncology; local laboratory capacity, professional education, patient advocacy and awareness, and supportive regulatory environments are also important determinants of whether molecular testing and matched treatment can be effectively delivered [36]. This is particularly relevant to the present study because it suggests that financial interventions alone may not fully address the implementation gap identified by participating specialists.

The finding concerning testing infrastructure is especially pertinent to the Bangladeshi context. These perceptions are consistent with recent assessments of cancer care in Bangladesh describing inadequate cancer-specific molecular genetics infrastructure and the need for some specimens to be sent outside the country for testing [3]. A recent assessment of cancer care in Bangladesh likewise described the lack of comprehensive cancer-specific molecular genetics testing facilities and reported that specimens may need to be sent abroad for molecular testing [3]. This provides important context for the prominence of testing-facility availability and turnaround time in the present survey. Expanding testing capacity may therefore have value beyond simply increasing the number of tests available; locally accessible and appropriately supported molecular diagnostics could potentially influence timeliness, continuity of care, and the feasibility of translating test results into treatment decisions.

Patient understanding and acceptance also emerged as an important non-financial dimension. This finding is consistent with evidence that patient knowledge, communication, beliefs, and acceptance can influence access to genomic testing and precision treatment. A multinational study of patients with breast cancer reported that cost and lack of understanding were among prominent perceived barriers to genetic testing, while respondents also identified updated clinical guidelines and public awareness initiatives among potential solutions [37]. Although germline genetic testing and tumor molecular profiling are not interchangeable, these findings illustrate the broader importance of patient understanding and communication within genomically informed cancer care. Improving laboratory availability without ensuring that patients understand the purpose, implications, and potential therapeutic consequences of molecular testing may therefore leave an important component of access unaddressed.

Other non-financial barriers, including limited local clinical guidelines, targeted therapy availability or supply issues, and physician awareness or training, were less frequently rated as major or very major barriers than cost and several infrastructure- or patient-related factors. This should not be interpreted as evidence that these factors are unimportant, particularly because this descriptive study was not designed to statistically rank barriers or determine their causal contribution. Instead, the findings illustrate the breadth of challenges that may influence implementation. International evidence similarly suggests that implementation of genomic medicine depends on coordinated organizational infrastructure, genomic training, testing policies, interpretation support, and access to treatment rather than any single component [16]. A recent roadmap addressing access to advanced biomolecular technologies similarly identifies price and availability alongside knowledge, infrastructure, clinical and laboratory operations, and evidence of value as interconnected challenges requiring coordinated solutions [38].

The generally favorable attitudes of respondents toward targeted therapy provide important context for interpreting these barriers. Overall, 80.8% agreed or strongly agreed that targeted therapy should become standard practice in Bangladesh where clinically indicated. Together with the relatively frequent recommendation of molecular profiling, this suggests a clinical environment broadly receptive to molecularly guided cancer care among the specialists surveyed. The findings therefore direct attention toward the practical conditions required to translate this clinical acceptance into treatment access, rather than attributing the implementation gap primarily to lack of professional support. Nevertheless, continued professional education and locally applicable guidance remain relevant as precision-oncology technologies, biomarkers, and therapeutic options continue to evolve.

Respondents’ proposed interventions closely reflected the principal barriers identified in the survey. Reduced testing cost was the most frequently selected strategy, followed by government-subsidized insurance coverage, emphasizing the perceived importance of affordability and financial protection. However, respondents also selected more local laboratories and increased drug availability, again demonstrating that improving access is not solely a financial issue. Financial protection mechanisms may be important in Bangladesh, where the broader cancer-care literature has documented substantial out-of-pocket expenditure and catastrophic financial consequences [34]. Contemporary precision-oncology literature similarly supports multidimensional approaches that combine improved affordability with strengthened clinical and laboratory infrastructure, awareness, operational capacity, and access to appropriate treatment [38].

The findings suggest several areas for policy consideration, including reducing the financial burden of molecular testing, developing government-supported or subsidized coverage mechanisms, expanding local molecular-testing capacity, improving turnaround times, strengthening access to clinically indicated targeted therapies, and developing locally applicable guidance for molecular testing and treatment selection. These measures should be considered potential priorities identified from clinicians’ perceptions rather than interventions whose effectiveness was established by the present study.

Quantitative evidence specifically characterizing cancer specialists’ perceptions of barriers to molecular profiling and targeted therapy in Bangladesh remains limited. The present study therefore provides an initial descriptive assessment of both financial and non-financial challenges perceived within clinical oncology practice. The cross-sectional design provides a snapshot of reported practices and perceptions at the time of the survey and does not permit assessment of temporal changes or causal relationships. Its findings should not be interpreted as establishing the relative causal importance of individual barriers, but rather as identifying areas requiring more comprehensive investigation. Larger multicenter studies involving a broader and more representative oncology workforce, together with patient-level measures of molecular testing, treatment access, affordability, turnaround time, and actual receipt of targeted therapy, will be important for validating these findings and identifying additional barriers not captured by the present survey. Such evidence could inform more comprehensive, context-specific strategies for improving access to precision oncology in Bangladesh.

### Limitations of the study

This study has several limitations. The relatively small sample size (N = 52) and non-probability sampling approach may limit the generalizability of the findings to the broader oncology workforce in Bangladesh. Nevertheless, given the specialized and relatively limited oncology workforce reported in published literature, the sample represents a considerable number of cancer specialists for an exploratory national survey. Respondents were predominantly clinical oncologists and were largely based in government hospitals, specialized cancer centers, and tertiary referral facilities, potentially limiting representation from other specialties and practice settings. The use of non-probability convenience sampling may have introduced selection bias, as specialists who were accessible through the investigators’ professional networks or more interested in precision oncology may have been more likely to participate. Consequently, the findings should not be interpreted as statistically representative of all cancer specialists in Bangladesh.

In addition, the findings reflect clinicians’ self-reported practices, perceptions, and estimates rather than objectively measured patient-level testing and treatment data and may therefore be subject to recall and reporting biases. The cross-sectional design also provides a snapshot of current practice and does not allow assessment of temporal changes or causal relationships. Despite these limitations, the study provides exploratory quantitative evidence on molecular profiling, targeted therapy receipt, and perceived barriers across multiple dimensions of precision oncology care in Bangladesh.

### Conclusion

This study highlights a substantial gap between molecular profiling and the reported receipt of targeted therapy among eligible patients in Bangladesh. Although financial constraints were the most prominent barriers, important non-financial challenges involving testing infrastructure, patient understanding and acceptance, and turnaround time were also identified. Improving precision oncology access will therefore require attention to barriers beyond financial constraints alone. These findings reflect cancer specialists’ perceptions and estimates rather than objectively measured patient-level utilization and should therefore be interpreted within the context of the study’s descriptive, non-probability survey design. Further larger, multicenter studies with more representative populations and patient-level data are needed to identify additional barriers and strengthen the evidence base for government-supported insurance coverage of molecular profiling and targeted therapies, as well as the development of stronger local guidelines to promote precision oncology–based cancer care in Bangladesh.

## Data Availability

The data supporting the findings of this study are available from the corresponding author upon reasonable request.
